# Integrated taxonomy reveals a new praying mantis species of *Phyllothelys* Wood-Mason, 1876 (Mantodea, Hymenopodidae) from Yunnan, China

**DOI:** 10.3897/BDJ.12.e132161

**Published:** 2024-10-04

**Authors:** Qinpeng Liu, Chao Wu, Bowen Ye, Xingyue Liu

**Affiliations:** 1 Department of Entomology, College of Plant Protection, China Agricultural University, Beijing, China Department of Entomology, College of Plant Protection, China Agricultural University Beijing China; 2 Key Laboratory of the Zoological Systematics and Evolution, Institute of Zoology, Chinese Academy of Sciences, Beijing, China Key Laboratory of the Zoological Systematics and Evolution, Institute of Zoology, Chinese Academy of Sciences Beijing China; 3 School of Future Design, Harbin Institute of Technology (Shenzhen campus), Shenzhen, China School of Future Design, Harbin Institute of Technology (Shenzhen campus) Shenzhen China

**Keywords:** Mantodea, *Phyllothelys*, new species, taxonomy, Yunnan

## Abstract

**Background:**

The genus *Phyllothelys* Wood-Mason, 1876 (Mantodea, Hymenopodidae) includes 22 species, with China being a major diversity hotspot, hosting 15 species in four groups.

**New information:**

A new species of this genus from Malipo, Yunnan, China, is described herein, namely *Phyllothelysqingjinum* **sp. nov.** A comprehensive taxonomic description, including geometric morphometric analysis, genetic distance and molecular phylogenetic analysis, were employed to illustrate its distinctiveness. This discovery fills a significant distribution gap for the *P.werneri* species group in southern Yunnan and emphasises the need for further exploration in this region.

## Introduction

The genus *Phyllothelys* Wood-Mason, 1876, classified within Hymenopodidae (Mantodea), comprises 22 species divided into four distinct species groups (Wu and Liu 2021). China represents a significant hotspot of species diversity for this genus, with 14 species across three groups exclusively reported from this region (Ehrmann and Roy 2009, Wu and Liu 2021). Notably, the *Phyllothelyswerneri* species group, which included six species prior to this study, exhibits a broad distribution across the mountainous areas south of the Yangtze River of China, extending from Medog in the west to Taiwan Island in the east (Karny 1915, Hua 1984, Zhou and Zhou 2004, Wu and Liu 2021). However, previous research identified southern Yunnan as a significant blank area for the distribution of this species group.

In this study, we describe a new species, *P.qingjinum* **sp. nov.** of *Phyllothelys* from Malipo, Yunnan of China based on molecular and morphological evidence. This discovery bridges the previously identified distribution gap of *P.werneri* species group in southern Yunnan, unveiling unrecognised diversity in this area. It also highlights the need for further comprehensive investigations into the diversity of *Phyllothelys* in southern Yunnan and the northern Indochina Peninsula.

## Materials and methods

### Taxonomy

Living specimens were collected at night by visual inspection using a head torch or under light traps. External genitalia were dissected in 10% potassium hydroxide (KOH) solution, clarified with pure water and subsequently stored in 70% ethanol in Eppendorf tubes for further research. Photographs were taken with a Nikon D7100 digital camera. All examined specimens are deposited in the collections of China Agricultural University, Beijing, China (CAU); the Institute of Zoology, Chinese Academy of Sciences, Beijing, China (IZCAS); the collection of Chao Wu in Beijing, China (CWC); and the collection of Bowen Ye in Shandong, China (CBW).

The classification system follows Schwarz and Roy (2019). Morphological terminology for adults and external male genitalia follows Brannoch et al. (2017) and Schwarz and Roy (2019).

### Morphometric analyses

We used the pronotum of 25 male specimens from six species within *Phyllothelyswerneri* species group as representatives to quantitatively assess and visualise the morphological differences between *P.qingjinum* sp. nov. and others (Table 1). The species *P.stigmosum* (Zhou & Zhou, 2004) was not considered in analysis due to the unavailability of the type specimen for examination and the possibility that it represents a synonym of *Phyllothelyswerneri*, based on certain aspects of the original description (Wu and Liu 2021). This species is also unlikely to be confused with *P.qingjinum* sp. nov. due to their distant distributions and significant differences in the pronotum (Zhou and Zhou 2004).

We employed tpsUtil 1.46 (Rohlf 2010) and tpsDig2 v.2.16 (Rohlf 2008) to extract and digitise eight homologous landmarks from the left margin of pronotum in each specimen (Suppl. material 1) (Brannoch et al. 2017). These landmark configurations were then scaled, translated and rotated against a consensus configuration using the Procrustes superimposition method (Bookstein 1991) in MorphoJ 1.06d (Klingenberg 2011). Finally, Principal Component Analysis (PCA) was performed on the reconfigured landmarks in MorphoJ 1.06d to assess variability in the shape space.

### Molecular phylogenetic analyses and genetic distance calculation

The partial Cytochrome Oxidase I (COI) sequence was selected as the molecular marker to conduct the phylogenetic analyses and genetic distance calculation. A total of 19 individuals were sampled for the analyses, covering three species groups in *Phyllothelys* (*P.werneri* species group, *P.sinense* species group and *P.westwoodi* species group), in which 17 of them are newly sequenced in this study and two are obtained from GenBank databases (Table 2).

For the newly-sequenced specimens, their genomic DNA was extracted from the thoracic and coxal muscle tissues by using the DNeasy Blood and Tissue kit (Qiagen, Hilden, Germany) and amplified using Ex Taq (TaKaRa, Tokyo, Japan). Primers for amplification are LCO-1490 (5’-GGTCAACAAATCATAAAGATATTGG-3’) and HCO-2198 (5’-TAAACTTCAGGGTGACCAAAAAATCA-3’) (Folmer et al. 1994). The PCR protocols implemented using a T100 thermal cycler (Bio-Rad, Hercules, CA, USA) were as follows: an initial 3-min denaturing step at 94°C; 35 cycles of 20 s at 94°C, 20 s at 50°C and 30 s at 72°C; with a final 5-min extension at 72°C. Sequences were bidirectionally sequenced using the same PCR primer pairs. Sequences alignment was carried out using ClustalW and edited using Geneious 10.1.3 (Kearse et al. 2012), generating a matrix with 620 nucleotide characters.

We employed both Maximum Likelihood (ML) and Bayesian Inference (BI) based on the alignment generated above. For the ML analyses, the best substitution model was determined as ‘TIM2+F+G4’ according to BIC (Bayesian information criterion scores) in ModelFinder (Kalyaanamoorthy et al. 2017) and the phylogenetic tree was constructed under the IQ-TREE v.1.6.12 (Trifinopoulos et al. 2016) using an ultrafast bootstrap approximation approach with 1000 bootstrap replicates. For the BI analyses, the best substitution model was determined in PartitionFinder2 v.2.1.1 (Lanfear et al. 2017) as ‘GTR+G+F’. Two simultaneous runs of two million generations were executed using MrBayes v.3.2.7 (Ronquist et al. 2012) for the dataset and trees were sampled every 1000 generations, with the first 25% discarded as burn-in. Stationarity was considered to be reached when the average standard deviation of split frequencies was below 0.01.

We used the same sequences alignment to calculate the genetic distances within *Phyllothelyswerneri* species group. The analysis was conducted in MEGA7 (Kumar et al. 2016) under the Kimura two-parameter (K2P) model and the result visualised in ggplot (package in R v.4.3.0 in RStudio for Windows).

## Taxon treatments

### Phyllothelys qingjinum

Liu, Wu, Ye, & Liu sp. nov.

6C95252C-55B0-5DE2-8EBD-3A40C1B9F834

2439DC24-2ED8-4D7E-9B3F-CC1A20D382B7

#### Materials

- **Type status:** Holotype. **Occurrence:** recordNumber: CAU; recordedBy: local collector; individualCount: 1; sex: male; lifeStage: adult; occurrenceID: 5D74CD2B-3CE8-5065-82D3-D63DE011F8D4 (Deposited in CAU).; **Location:** country: China; stateProvince: Yunnan; locality: Wenshan Zhuang Miao Autonomous Prefecture, Malipo, Xiajinchang, Yunling village; verbatimElevation: 1391 m; verbatimCoordinates: 23°9′26″N, 104°51′14″E; **Event:** eventDate: 10-12-23
- **Type status:** Paratype. **Occurrence:** recordNumber: CAU; recordedBy: Danyang Zhou; individualCount: 1; sex: male; lifeStage: adult; occurrenceID: 409EB288-DFC4-5C3D-9209-62397E320481 (Deposited in CAU).; **Location:** country: China; stateProvince: Yunnan; locality: Wenshan Zhuang Miao Autonomous Prefecture, Malipo, Xiajinchang, Jinkuxian; verbatimElevation: 1403 m; verbatimCoordinates: 23°11′17″N; 104°49′7″E; **Event:** eventDate: 08-07-23
- **Type status:** Paratype. **Occurrence:** recordNumber: CAU; recordedBy: local collector; individualCount: 3; sex: male; lifeStage: adult; occurrenceID: 1B3263FF-7658-5124-8D69-4E8E25ED748C (Deposited in CAU).; **Location:** country: China; stateProvince: Yunnan; locality: Wenshan Zhuang Miao Autonomous Prefecture, Malipo, Xiajinchang, Yunling village; verbatimElevation: 1391 m; verbatimCoordinates: 23°9′26″N, 104°51′14″E; **Event:** eventDate: 10-12-23
- **Type status:** Paratype. **Occurrence:** recordNumber: IZCAS; recordedBy: local collector; individualCount: 3; sex: male; lifeStage: adult; occurrenceID: A7F2E489-7FA6-532B-B02C-873EDF930874 (Deposited in IZCAS).; **Location:** country: China; stateProvince: Yunnan; locality: Wenshan Zhuang Miao Autonomous Prefecture, Malipo, Xiajinchang, Yunling village; verbatimElevation: 1391 m; verbatimCoordinates: 23°9′26″N, 104°51′14″E; **Event:** eventDate: 10-12-23
- **Type status:** Paratype. **Occurrence:** recordNumber: CWC; recordedBy: local collector; individualCount: 2; sex: male; lifeStage: adult; occurrenceID: 93B1321C-D238-5533-8D96-EB23C5671E12 (Deposited in CWC).; **Location:** country: China; stateProvince: Yunnan; locality: Wenshan Zhuang Miao Autonomous Prefecture, Malipo, Xiajinchang, Yunling village; verbatimElevation: 1391 m; verbatimCoordinates: 23°9′26″N, 104°51′14″E; **Event:** eventDate: 10-12-23
- **Type status:** Paratype. **Occurrence:** recordNumber: CBW; recordedBy: local collector; individualCount: 1; sex: male; lifeStage: adult; occurrenceID: 21778A28-5905-593C-BDC3-8A1534C2CA27 (Deposited in CBW).; **Location:** country: China; stateProvince: Yunnan; locality: Wenshan Zhuang Miao Autonomous Prefecture, Malipo, Xiajinchang, Yunling village; verbatimElevation: 1391 m; verbatimCoordinates: 23°9′26″N, 104°51′14″E; **Event:** eventDate: 10-12-23

#### Description

**Figure 1.**
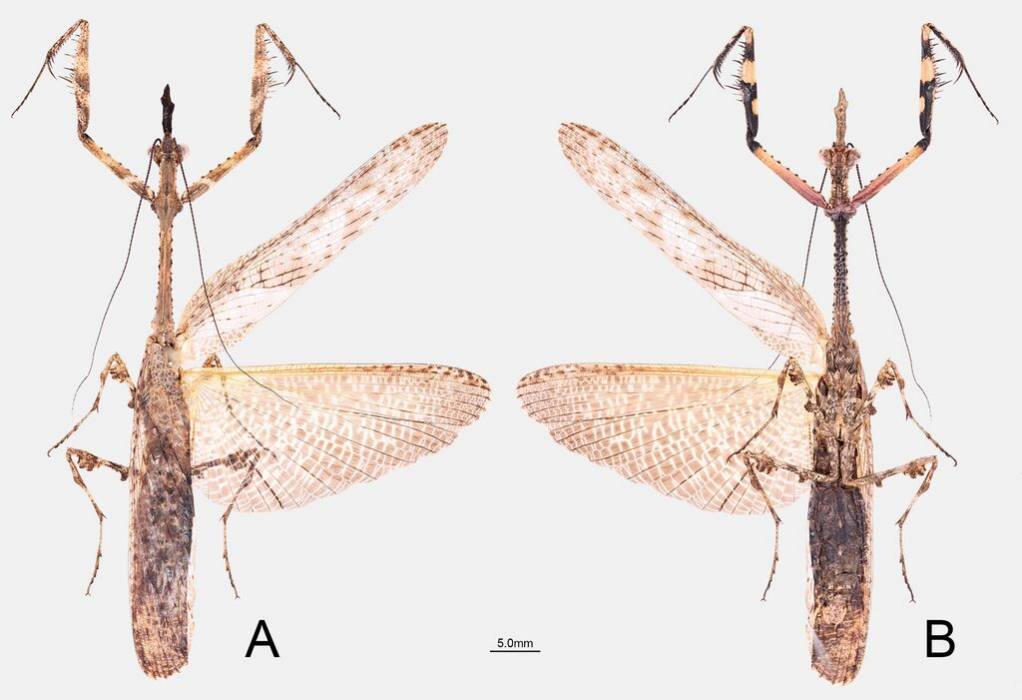
Holotype of *P.qingjinum* sp. nov. **A** Dorsal view; **B** Ventral view.

**Description of male.** Medium-sized *Phyllothelys*, similar to *P.werneri*, but body small-sized, lateral pronotal expansion of pronotum comparatively large, subtriangular (Fig. 1).

**Figure 2.**
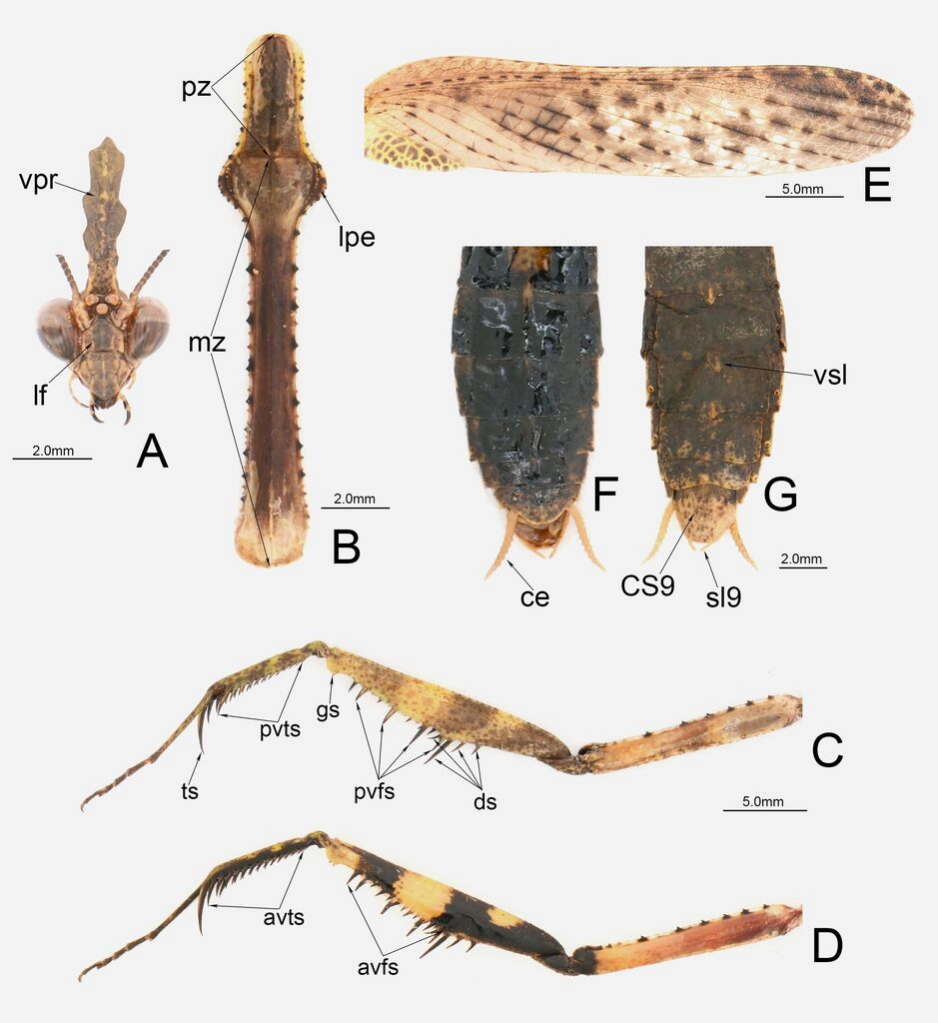
Body annotation of *P.qingjinum* sp. nov. **A** Head, frontal aspect; **B** Pronotum, dorsal view; **C** Prothoracic leg, dorsal view. **D** Prothoracic leg, ventral view; **E** Fore wing; **F** Abdomen, dorsal view; **G** Abdomen, ventral view. Abbreviations: vpr = vertical process; lf = lower frons; pz = prozone; lpe = lateral pronotal expansion; mz = metazone; ts = tibial spur; pvfs = posteroventral femoral spines; gs = genicular spur; pvts = posteroventral tibial spines; ds = discoidal spines; avfs = anteroventral femoral spines; avts = anteroventral tibial spines; vsl = vertical sternite lobe; ce = cercus; CS9 = ♂ coxosternite; sl9 = stylus.

**Head.** Triangular. Eyes rounded, anteriorly protruding. Ocellar tubercle flat, higher than compound eyes; ocelli elliptical, larger, central ocellus slightly smaller than lateral ocelli. Lower frons sub-pentagonal, about as high as wide, with smooth surface, with prominent top and with obtuse-angle upper margin; clypeus wider than high, with median keel. Vertex with long vertical process, dorsally black, with inflated base and a median keel; vertical process narrow, slightly longer than head, with 2–3 obtuse angular lobes on wavy lateral margin and blunt apex. Antennae filiform, longer than pronotum, but not exceeding body length (Fig. 2A).

**Pronotum.** Long and very slender, narrower than head width. Cross section of metazona triangular, dorsal surface of metazona keeled along its mid-line; supracoxal dilation distinct, with obviously lateral pronotal expansion, subtriangular, but without sharp margin, lateral margin with small spines. Lateral margin of pronotum with sparsely arranged black large grainy spines, interleaved small ones; area around large spines on lateral margin black. Ratio of pronotum length to supracoxal dilation width about 4.76–4.90; ratio of metazona length to prozona length about 3.29–3.33. Prosternum flat, brownish, with dense black spots (Fig. 2B).

**Prothoracic legs.** Elongate; coxae shorter than metazona of pronotum, with about 6–7 spine-like tubercles on anterior margin, black, interleaved with 2–3 small spines; femora slightly longer than coxae, dorsal margin straight, with a series of sparsely small denticles; femora with 4 posteroventral femoral spines, 4 discoidal spines and 14–15 anteroventral femoral spines, amongst which the first anteroventral femoral spine at distal end is larger than other spines, the spination pattern of which is iIiIiIiIiIiIiiI, posteroventral femoral spines elongate, vertical, with apex slightly bent backwards; tibiae with 12–13 posteroventral tibial spines and 14–15 anteroventral tibial spines, posteroventral and anteroventral spines oblique, gradually lengthening distad. Tarsus slightly longer than tibia, first segment of tarsus longer than combined length of remaining segments (Fig. 2C, D).

**Meso- and metathoracic legs.** Slender, slightly elongate. Femora with disjunctive proximal and pre-apical lobes; proximal lobe observably smaller than pre-apical lobe, pre-apical lobe large, semicircular, but with irregular edge. Tibiae shorter than femora; slender, middle of tibiae inflated, becoming slender apicad. Tarsus slightly longer than tibia, first joint of tarsi longer than combined length of remaining segments.

**Wings.** Forewing long and narrow, subhyaline; apex round; costal area narrow, with small black spots; discoidal area with transparent cells, but more cells smoky; stigma completely reduced. Hind wing wide, shorter than forewing; significantly smoky, subhyaline, except costal field; apex of costal field with black spots (Fig. 2E).

**Abdomen.** Abdomen long and narrow, flat, slightly fusiform; with small prominent lateral lobes on tergites 4–6, unobtrusive; sternite 3–6 with vertical lobe, triangular, slightly blunt at tip, with a median carina, the largest one present on sternite 5. Supra-anal plate transverse, with a rounded posterior edge; cerci hairy, slightly flattened, with a conical terminal joint. Male subgenital plate trapezoidal, about as long as wide, with short styli (Fig. 2F, G).

**Figure 3.**
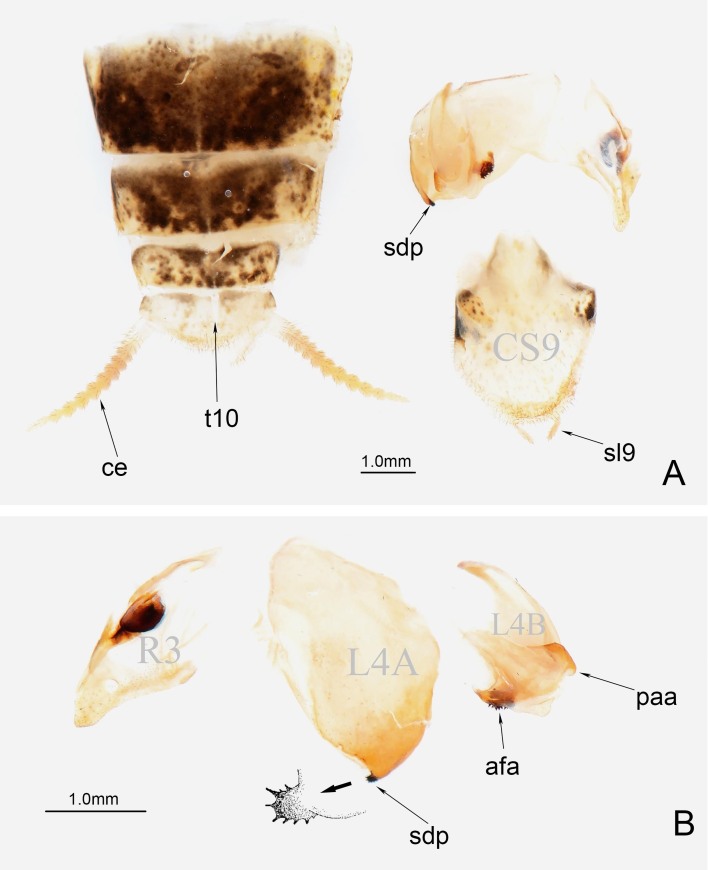
Male external genitalia of *P.qingjinum* sp. nov. **A** General feature of genital segments; **B** Detail feature of phallic complex. Abbreviations: ce = cercus; t10 = tergite 10 (= supra-anal plate); sdp = secondary distal process; sl9 = stylus; afa = anterior process (left phallomere); paa = posterior process (left phallomere).

**External genitalia.** Weakly sclerotised. Ventral phallomere broad, roughly rhomboidal, significantly pigmented on its lower left side edge; left margin of ventral phallomere weakly sclerotised; lateral secondary distal process small, strongly sclerotised, with numerous spines. L4B of left phallomere broad, paa of left phallomere short, digitiform; afa broad, saddle-shaped, strongly sclerotised, with numerous sharp spines (Fig. 3).

**Figure 4.**
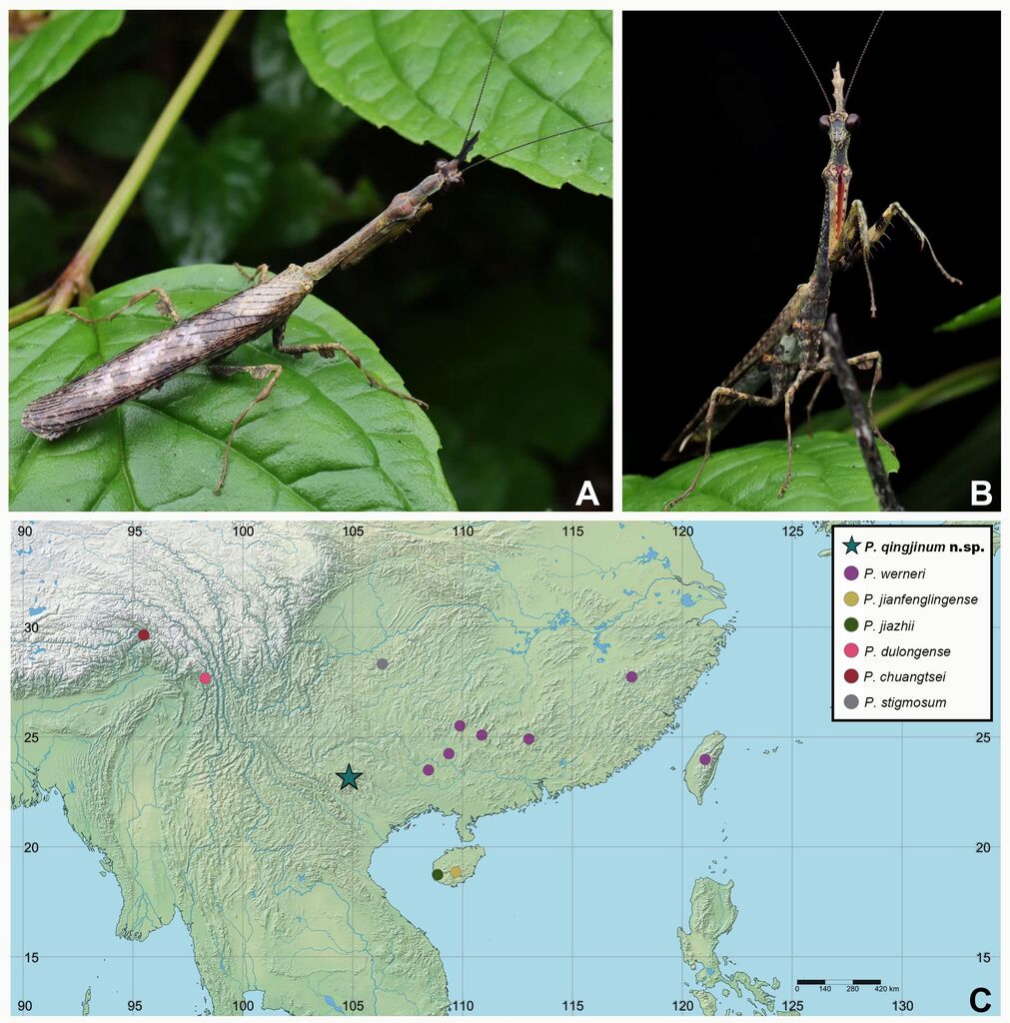
Living habitus and distribution of *P.qingjinum* sp. nov. **A, B** Living habitus by Danyang Zhou (Ziyang, Sichuan, China), published with permission; **C** Distribution of *P.qingjinum* sp. nov. and other species in *P.werneri* species group, created under SimpleMapper (https://www.simplemappr.net. Accessed 07 June 2024).

**Colouration.** Body entirely brown, slightly moss-coloured when alive (Fig. 4A, B). Antennae blackish-brown. Dorsal portion of cephalic vertical process black. Interior surface of fore coxae reddish-brown, distally positioned with black area, tubercles on anterior margin black; interior surface of fore femora yellowish-brown, black markings present at base, middle and apex on interior margin, amongst which the former two black markings are united; femoral spines black, except that located on the brown part. Spines on lateral margin of pronotum black; prosternum brownish, scattered with black spots. Forewing blackish-brown, with some smoky stains; translucent, except some cells on distal half completely transparent. Hind wing translucent, blackish-brown, with yellow costal field, discoidal area with yellowish veins. Abdomen ventrally brown, dorsally shiny black; subgenital plate yellowish-brown with black spots, styli and cerci yellowish-brown.

**Measurements (length in mm).** Body (head to abdomen end): 52.75–54.95; body (head to wings end): 58.60–62.14; pronotum: 15.30–17.76; prozona: 3.46–3.95; metazona: 11.84–13.88; fore coxa: 8.39–10.64; fore femur: 9.36–11.46; fore tibia: 5.55–7.02; hind femur: 7.82–9.35; hind tibia: 6.82–8.54; forewing: 32.03 –36.79; hind wing: 28.70–33.16.

**Distribution.** China (Yunnan) (Fig. 4C).

**Etymology.** The species name is derived from the Chinese word ‘青衿’ (Qingjin), which refers to the green cross-collared deep robe in ancient Hanfu. This term metaphorically refers to the distinct green colouration on the dorsal edge of the pronotum of this species when it is alive.

## Identification Keys

### Key to species of *Phyllothelyswerneri* species group, male

**Table:** 

1	Pronotum very slender, ratio of pronotum length to supracoxal dilation width greater than 6; occurs in Hainan, China	2
–	Ratio of pronotum length to supracoxal dilation width less than 6	3
2	Fore tibia with 12–13 posteroventral tibial spines; ratio of pronotum length to supracoxal dilation width about 6.5	*Phyllothelysjianfenglingense*
–	Fore tibia with only 8–9 posteroventral tibial spines; ratio of pronotum length to supracoxal dilation width about 6.2	*Phyllothelysjiazhii*
3	Pronotum without obvious lateral pronotal expansion; ratio of pronotum length to supracoxal dilation width greater than or equal to 5.5	4
–	Supracoxa dilation expressed with prominent lateral pronotal expansion; ratio of pronotum length to supracoxal dilation width about 4.8–5.1	5
4	Body length (head to wings end) about 62.5–64.2; ratio of pronotum length to supracoxal dilation width about 5.5-5.6; occurs in southeast China	*Phyllothelysdulongense*
–	Body length (head to wings end) about 62.5–64.2; ratio of pronotum length to supracoxal dilation width about 5.5-5.6; occurs in southeast China	*Phyllothelyswerneri*
5	Numerous longer sharp spines on sdp and afa of male genitalia; occurs in southeast Yunnan China	*Phyllothelysqingjinum* sp. nov.
–	Lacking long sharp spines on sdp afa covered by short spines; occurs in Medog of China	*Phyllothelyschuangtsei*

## Analysis

### Geometric morphometrics

**Figure 5.**
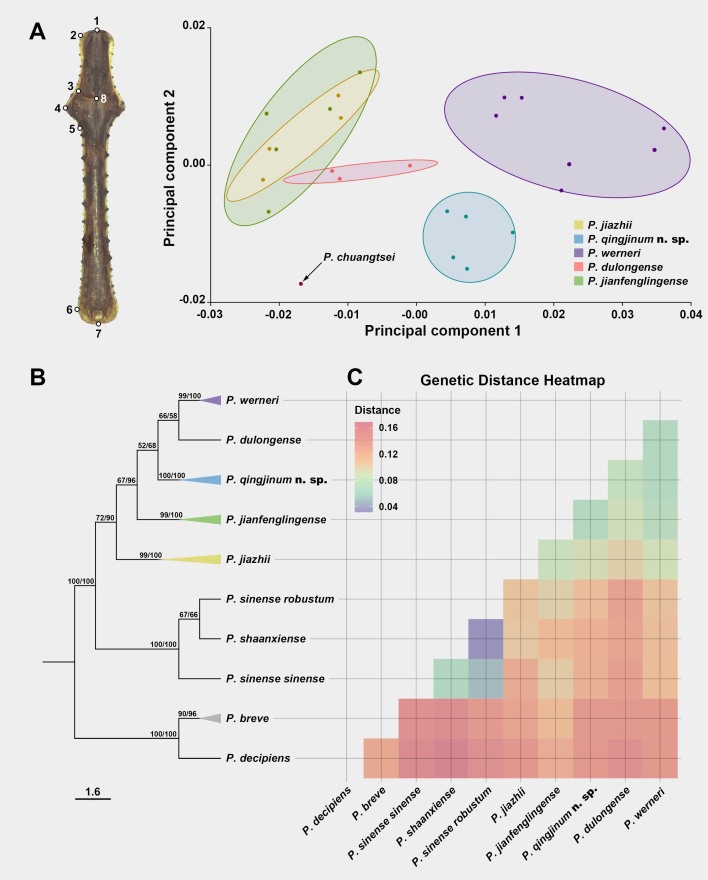
Morphological and molecular divergence of *P.qingjinum* sp. nov. relative to other species in the *P.werneri* group. **A.** Eight morphological landmarks on the pronotum used for Principal Component Analysis (PCA) (the left) and the resulting pronotum shape differences amongst species shown on the right, with circles are 80%-equal frequency ellipses of each species. **B.** Phylogenetic relationships within the *P.werneri* group, constructed using IQ-TREE and MrBayes, feature a cladogram-based framework from IQ-TREE, with branches of multiple samples from the same species collapsed into coloured triangles. Node support values are noted directly beside each node, formatted as Bootstrap value/Bayesian posterior probability (%). **C.** Heatmap illustrates pairwise genetic distances between species under K2P model, where the intensity of the colour reflects the degree of genetic distance, with deeper reds indicating greater genetic divergence.

Based on the morphometrics of the pronotum, we compared five previously-described species to *P.qingjinum* sp. nov. in the *P.werneri* species group (Fig. 5A). The contribution of the first principal (PC1) component accounted for 62.21% of the total variation, whereas the second principal (PC2) account for 13.92%. In the factorial map, the points representing *P.qingjinum* sp. nov. were far away from the others. The points of *P.chuangtsei* and *P.werneri* were also each distinctly separated from other species. In contrast, the points representing *P.jiazhii*, *P.jianfenglingense* and *P.dulongense* were closer to each other, with their 80%-equal frequency ellipses showing considerable overlap.

### Phylogenetic analyses and genetic distance

Phylogenetic analysis using MrBayes and IQ-TREE revealed identical topologies and generally high support values (Fig. 5B). The results supported the monophyly of all sampled species and species groups and indicated that the *P.westwoodi* species group is the sister group to the clade (*P.sinense* species group + *P.werneri* species group). The phylogenetic position of *P.qingjinum* sp. nov. is recovered to be within the *P.werneri* species group, as the sister to the clade (*P.werneri* + *P.dulongense*).

The results of the genetic distance analysis showed that the average genetic distance between species in the genus *Phyllothelys* was 0.11 (Fig. 5C). Specifically, the average genetic distances between *P.qingjinum* sp. nov. and the *P.westwoodi* species group, *P.sinense* species group and other species within the *P.werneri* species group were 0.14, 0.12 and 0.08, respectively. Notably, the genetic distance between *P.qingjinum* sp. nov. and *P.jianfenglingense* was the smallest amongst all comparisons, at 0.07, which is still significantly lower than the average interspecific distance within the genus (0.11).

## Discussion

### Species group assignment

The posteroventral femoral spines of *P.qingjinum* sp. nov. are elongate, with their length nearly equal to the width of femora and apex slightly bending backwards (Fig. 1). In the interior surface of prothoracic femora, the basal and middle dark spots merge on the basal half (Fig. 2D). These characteristics align with the key diagnostic features of the *P.werneri* species group as described by Wu and Liu (2021), while they are different from the other three species groups. In terms of molecular evidence, *P.qingjinum* sp. nov. and species within the *P.werneri* species group exhibit relatively low genetic differentiation (average 0.08) compared to the *P.sinense* species group (average 0.12) and the *P.westwoodi* species group (average 0.14). Phylogenetic analyses using different methods consistently nest *P.qingjinum* sp. nov. within the *P.werneri* species group. Therefore, based on the morphological characteristics of the posteroventral femoral spines and the DNA barcoding evidence, we infer that *P.qingjinum* sp. nov. should be included in the *P.werneri* species group.

### Comparison with other Phyllothelys species

*P.qingjinum* sp. nov. can be easily distinguished from other species in the genus *Phyllothelys* by the morphology and coloured patches of the prothoracic legs (Fig. 2D). The densely arranged large colour patches in the discoidal area of the forewings of *P.qingjinum* sp. nov. resemble those of *P.jianfenglingense* and *P.dulongense*, both of which also belong to the *P.werneri* species group (Fig. 2E). However, *P.qingjinum* sp. nov. present a comparatively prominent supracoxal dilation with obvious lateral pronotal expansion, which sets it apart from these two species (Fig. 2B; Suppl. material 1). Within the *P.werneri* species group, only *P.chuangtsei*, aside from the new species described here, exhibits a developed lateral pronotal expansion around supracoxa dilation. However, *P.qingjinum* sp. nov. can be further distinguished from *P.chuangtsei* by its larger size and the presence of numerous longer, sharp spines on the sdp and afa of the male external genitalia (Fig. 3B).

## Supplementary Material

XML Treatment for Phyllothelys qingjinum

2FC91950-6ACB-5F76-9E5A-692DB7FDA8E6 10.3897/BDJ.12.e132161.suppl1 Supplementary material 1 TPS file and figures of pronotum Data type morphological Brief description TPS file and figures of pronotum used in morphometric analyses. File: oo_1092609.rar https://binary.pensoft.net/file/1092609 Qinpeng Liu, Chao Wu, Bowen Ye, Xingyue Liu

## Figures and Tables

**Table 1.** Specimens used in morphometric analyses.

**Species**	**Specimen Identifier**	**Collecting Information or Reference**
*P.chuangtsei*	NA	Wu and Liu (2021) fig. 7O
*P.dulongense*	NA	Wu and Liu (2021) fig. 5D
	NA	Wu and Liu (2021) fig. 7N
	CWCP162	China, Yunnan, Gongshan, Dulongjiang, 18-VII-2019, Chao Wu
*P.jianfenglingense*	CAUP139	China, Hainan, Ledong, Jianfeng Mountain, VI-2021, Haoming Zang
	CBW1	China, Hainan, Ledong, Jianfeng Mountain, 19-VII-2022, local collector
	CBW2	China, Hainan, Ledong, Jianfeng Mountain, 19-VII-2022, local collector
	NA	Wu and Liu (2021) fig. 5B
	NA	Wu and Liu (2021) fig. 7M
*P.jiazhii*	CAUP11	China, Hainan, Ledong, Jianfeng Mountain, 24-X-2017, Jianyun Wang
	CAUP129	China, Hainan, Ledong, Jianfeng Mountain, 2019, Zeyi Lyu
	NA	Wu and Liu (2021) fig. 5C
	NA	Wu and Liu (2021) fig. 7K
*P.werneri*	CAUP77	China, Fujian, Sangang, 30-VIII-1987
	CAUP67	China, Chongqing, Simian Mountain, 14-VIII-2023, local collector
	CAUP2	China, Guangxi, Nanning, Daming Mountain, 14-IX-2022, local collector
	CAUP16	China, Sichuan, Luzhou, Gulin, 3-VIII-2022, local collector
	CAUP138	China, Guizhou, Bijie, VII-2023, local collector
	CAUP118	China, Guangxi, Nanning, Daming Mountain, 1-IX-2019, Mingxia Gong
	CAUP105	China, Fujian, Nanping, Jianyang, 5--X-2023, local collector
*P.qingjinum* sp. nov.	CAUP76	China, Yunnan, Wenshan, Malipo, VIII-2023, Danyang Zhou
	CAUP89	China, Yunnan, Wenshan, Malipo, X-2023, local collector
	CAUP90	China, Yunnan, Wenshan, Malipo, X-2023, local collector
	CAUP93	China, Yunnan, Wenshan, Malipo, X-2023, local collector
	CAUP96	China, Yunnan, Wenshan, Malipo, X-2023, local collector

NA, not available.

**Table 2.** Specimen used in phylogenetic analyses and genetic distance.

**Species**	**Specimen Identifier**	**Accession Number**	**Collecting Information**
*P.werneri*	CAUP54	PQ032523	China, Taiwan, Jiayi, 1-VII-2019, Mingcen Wang
	CAUP10	PQ032528	China, Taiwan, Nantou, Lugu, 3-XI-2016, Yisheng Zhao
*P.dulongense*	CWCP162	PQ032526	China, Yunnan, Gongshan, Dulongjiang, 18-VII-2019, Chao Wu
*P.qingjinum* sp. nov.	CAUP96	PQ032521	China, Yunnan, Wenshan, Malipo, X-2023, local collector
	CAUP89	PQ032522	China, Yunnan, Wenshan, Malipo, X-2023, local collector
	CAUP76	PQ032519	China, Yunnan, Wenshan, Malipo, VIII-2023, Danyang Zhou
*P.jianfenglingense*	CAUP46	PQ032520	China, Hainan, Ledong, Jianfeng Mountain, 19-VII-2022, local collector
	CAUP52	PQ032529	China, Hainan, Ledong, Jianfeng Mountain, 18-VII-2022, Huaiyu Liu
	CAUP85	PQ032518	China, Hainan, Ledong, Jianfeng Mountain, local collector
*P.jiazhii*	CAUP129	PQ032524	China, Hainan, Ledong, Jianfeng Mountain, 2019, Zeyi Lyu
	CAUP3	PQ032527	China, Hainan, Ledong, Jianfeng Mountain, V-2022, Mingyuan Fan
	CAUP11	PQ032530	China, Hainan, Ledong, Jianfeng Mountain, 24-X-2017, Jianyun Wang
	CAUP128	PQ032525	China, Hainan, Wuzhishan, Wuzhi Mountain, 17-III-2023, local collector
*P.breve*	NA	MT024239	NA
	CAUP37	PQ032517	Vietnam, Dak Lak, Yok Don National Park, 9-V-2012, Xingyue Liu
*P.decipiens*	NA	FJ802898	NA
*P.shaanxiense*	CAUP35	PQ032515	China, Shaanxi, Xi’an, Zhuque Forestry Park, 2021, Chuxiang Zhao
*P.sinenserobustum*	CAUP44	PQ032516	China, Henan, Nanyang, Baotianman, 25-VII-2022, local collector
*P.sinensesinense*	CAUP33	PQ032514	China, Zhejiang, Hangzhou, Tianmu Mountain, 30-VIII-2020, local collector

* indicates the sequences generated in this study; NA, not available.
